# The Physiological Role of Mitophagy: New Insights into Phosphorylation Events

**DOI:** 10.1155/2012/354914

**Published:** 2012-03-07

**Authors:** Yuko Hirota, Dongchon Kang, Tomotake Kanki

**Affiliations:** Department of Clinical Chemistry and Laboratory Medicine, Kyushu University Graduate School of Medical Sciences, 3-1-1 Maidashi, Higashi-ku, Fukuoka 812-8582, Japan

## Abstract

Mitochondria play an essential role in oxidative phosphorylation, fatty acid oxidation, and the regulation of apoptosis. However, this organelle also produces reactive oxygen species (ROS) that continually inflict oxidative damage on mitochondrial DNA, proteins, and lipids, which causes further production of ROS. To oppose this oxidative stress, mitochondria possess quality control systems that include antioxidant enzymes and the repair or degradation of damaged mitochondrial DNA and proteins. If the oxidative stress exceeds the capacity of the mitochondrial quality control system, it seems that autophagy degrades the damaged mitochondria to maintain cellular homeostasis. Indeed, recent evidence from yeast to mammals indicates that the autophagy-dependent degradation of mitochondria (mitophagy) contributes to eliminate dysfunctional, aged, or excess mitochondria. In this paper, we describe the molecular processes and regulatory mechanisms of mitophagy in yeast and mammalian cells.

## 1. Selective Degradation of Mitochondria by Autophagy

 Autophagy is a catabolic process that degrades cytoplasmic components and organelles and is conserved in almost all eukaryotes. Autophagy is initiated in response to cellular stresses such as nutrient starvation, oxidative stress, infection, or inflammatory stimuli. Upon its induction, a cup-shaped double-membrane structure, called an isolation membrane (or phagophore), emerges in the cytoplasm, then the isolation membrane elongates with curvature and finally becomes enclosed, forming an autophagosome containing cytoplasmic components. Subsequently, autophagosomes fuse with lysosomes/vacuoles, and lysosomal hydrolases degrade the sequestered material [1–5]. This process facilitates physiological processes such as survival during starvation, clearance of dysfunctional or aggregated proteins and organelles, development, differentiation, and aging [6–8]. In addition to the nonselective degradation of cytoplasmic components, autophagy can selectively degrade specific organelles or proteins. These include peroxisomes, endoplasmic reticulum, ribosomes, the nucleus, intracellular pathogens, protein aggregates, lipid droplets, and secretory granules. These catabolic processes are termed pexophagy, reticulophagy (ERphagy), ribophagy, nucleophagy, xenophagy, aggrephagy, lipophagy, and zymophagy, respectively. Similarly, the yeast Cvt complex (a protein complex comprising aminopeptidase I (Ape1) and alpha-mannosidase (Ams1)) is delivered to vacuoles *via* an autophagy-like process; Ape1 and Ams1 are processed and activated in the vacuoles, and this autophagic process is called the Cvt pathway. It has been known for some time that mitochondria are also degraded by autophagy in mammalian cells (first described by Clark in 1957 [9]) and in yeast (first described by Takeshige and colleagues in 1992 [10]), but this selective autophagic process has recently been described in more detail. Daughter mitochondria with reduced membrane potential after a fission event are preferentially removed by autophagy in mammalian cells [11]. Photoirradiation-damaged mitochondria are selectively degraded by autophagy in hepatocytes [12, 13]. During the maturation of erythroid cells, mitochondria are preferentially degraded by autophagy in a manner dependent on the mitochondrial outer membrane protein Nix [14, 15]. Recently, it has been reported that there are two types of autophagy in mammalian cells: autophagy related protein 5 (Atg5) and Atg7-dependent (conventional) autophagy and Atg5/Atg7-independent (alternative) autophagy [16]. Both conventional and alternative autophagic processes are implicated in the autophagic degradation of mitochondria during erythroid cell maturation [16, 17]. Similarly, during white adipose tissue differentiation, mitochondria are preferentially degraded by autophagy [18]. When yeast cells were cultured in lactate-containing medium as the sole carbon source and were subjected to nitrogen starvation, the mitochondria were exclusively taken into microautophagic structures [19]. These findings support the idea that mitochondria are selectively recognized and degraded by autophagy. The identification of the yeast mitophagy-specific protein Atg32, which plays a key role in the recognition of mitochondria by the autophagic machineries, confirmed the existence of selective degradation of mitochondria by autophagy [20, 21].

## 2. The Mechanisms of Selective Autophagy of Mitochondria in Yeast

 Atg11 is a cytosolic adaptor protein that is required for selective cargo recognition by autophagy. For example, during the Cvt pathway, the cargo proteins Ape1 and Ams1 generate a complex with the receptor protein Atg19 that is recognized and bound by Atg11. Similarly, during pexophagy, *Pichia pastoris* Atg30 (PpAtg30) binds peroxisomal proteins PpPex3 and PpPex14 and is recognized and bound by Atg11. Finally, in both cases, Atg11 transports the cargo to the pre-autophagosomal structure/phagophore assembly site (PAS), where the isolation membrane emerges, and the cargo is surrounded by the autophagosome [22, 23]. Atg11 is also essential for mitophagy, suggesting the presence of a receptor protein for mitophagy that corresponds to Atg19 or PpAtg30 in the Cvt pathway and pexophagy, respectively, [24]. A genetic screen for yeast mutants defective in mitophagy identified such a receptor protein, which is now known as Atg32 [20, 21, 25]. Atg32 is a mitophagy-specific protein that is not required for nonselective autophagy or other types of selective autophagy [20, 21]. Atg32 consists of 529 amino acids and localizes in the mitochondrial outer membrane with its N-terminal domain towards the cytoplasm. Similarly to the Cvt pathway and pexophagy, when mitophagy is induced, Atg32 is bound by Atg11 and the Atg11–Atg32 complex recruits mitochondria to the PAS [20, 21]. During this recruitment step, Atg32 interacts with Atg8 *via* its WxxI motif. This Atg32–Atg8 interaction is thought to increase the efficiency of mitochondrial sequestration by the isolation membrane [20].

## 3. Regulation of Mitophagy in Yeast

 Although the molecular processes by which the autophagic machinery selects and degrades mitochondria have been revealed, little is known about the upstream signaling pathways. Recently, it was reported that the related signaling pathways of two mitogen-activated protein kinases (MAPKs), Slt2 and Hog1, are involved in the induction of mitophagy [26, 27]. In the Slt2 signaling pathway, all of protein kinase C (Pkc1), MAPKKK (Bck1), MAPKK (Mkk1/Mkk2), Slt2, and the upstream cell surface stress sensor Wsc1 are required for mitophagy [26]. In the Hog1 signaling pathway, Pbs2-Hog1 and the upstream stress sensor Sln1 are required for mitophagy [26]. The downstream proteins in both pathways have not been identified. The role of Slt2 is, however, controversial: in the above-mentioned study, nitrogen starvation-induced mitophagy was deficient in *slt2*-deleted cells [26], whereas another study reported normal mitophagy in *slt2*-deleted cells cultured to the post-log phase [28]. The Slt2-related signaling pathway might be associated with starvation-induced mitophagy only.

 Recently, we found that, when mitophagy is induced, Ser114 and Ser119 on Atg32 are phosphorylated and that the phosphorylation of Atg32, especially on Ser114, mediates the Atg32–Atg11 interaction and mitophagy [27]. Similarly it has been noted that phosphorylation of Ser112 on PpAtg30 is required for PpAtg30–PpAtg11 interaction and pexophagy in *Pichia pastoris *[21]. These findings suggest that both mitophagy and pexophagy are regulated by kinase activity and/or the localization of the kinases that phosphorylate Atg32 and/or PpAtg30. The kinase(s) that directly phosphorylate Atg32 or PpAtg30 have not been identified. Although the MAPK Hog1 is required for Atg32 phosphorylation, the direct phosphorylation of Atg32 by Hog1 was not observed in an *in vitro* phosphorylation assay [27]. Presumably, the unidentified kinase that phosphorylates Atg32 is downstream of Hog1 and Slt2.

 Atg33 is a mitophagy-related protein that was identified by a genetic screen for yeast mutants defective in mitophagy [25]. Atg33 is located in the mitochondrial outer membrane and functions in mitophagy but not in nonselective autophagy, the Cvt pathway, or pexophagy. Interestingly, in an *atg33*-knockout strain, although mitophagy was partially inhibited when induced by starvation, it was blocked almost completely when induced during the stationary phase. Although the function of Atg33 in mitophagy is unknown, it might be a factor for the selection or detection of damaged or aged mitochondria when cells have reached the stationary phase [25, 29]. Further studies are required to reveal the function of Atg33 in mitophagy.

In addition to Atg33, Whi2, Uth1, and Aup1 have also been reported as related to mitophagy [30–32]. Whi2 is a stress response protein that predominantly influences mitophagy and, to a lesser extent, autophagy [31]. Müller and Reichert speculated that Whi2 and the Ras/PKA (protein kinase A) signaling pathway are linked to the regulation of mitophagy [33]. Uth1 is a mitochondrial outer membrane protein and is reported to be required for mitophagy induced by rapamycin or nitrogen starvation [32]. Aup1 was identified by a screen for protein phosphatase homologs that interact with the serine/threonine kinase Atg1 that is required for autophagy and is suggested to be needed for efficient mitophagy to survive in prolonged stationary phase culture in a medium containing lactate as the carbon source [30]. Interestingly, it was shown that deletion of *RTG3*, a transcription factor that mediates the retrograde signaling pathway, causes a defect in stationary phase mitophagy and that deletion of *AUP1* leads to alterations in the patterns of Rtg3 phosphorylation under these conditions, implying that the function of Aup1 in mitophagy may be regulation of Rtg3-dependent transcription [34]. Inconsistently, both Uth1 and Aup1 have also been reported to be not required for mitophagy [21] and were not identified in genome-wide mitophagy screens [20, 29]. Further studies are required to clarify these discrepancies, which could be due to differences in the condition used to assess autophagy.

 Cellular oxidative status is one factor that contributes to the induction of mitophagy. Deffieu et al. reported that N-acetylcysteine, which increases cellular levels of reduced glutathione, prevents mitophagy [35]. Okamoto et al. reported that the expression of Atg32 is suppressed by N-acetylcysteine treatment, and, as a result, mitophagy is inhibited [20]. These findings suggest that Atg32 expression and mitophagy are affected by cellular oxidative conditions. Because mitophagy is thought to preferentially eliminate damaged mitochondria, it is reasonable that cellular oxidative status, which is compromised by reactive oxygen species (ROS) generated by damaged mitochondria, is related to the induction of mitophagy.

**Figure 1:**
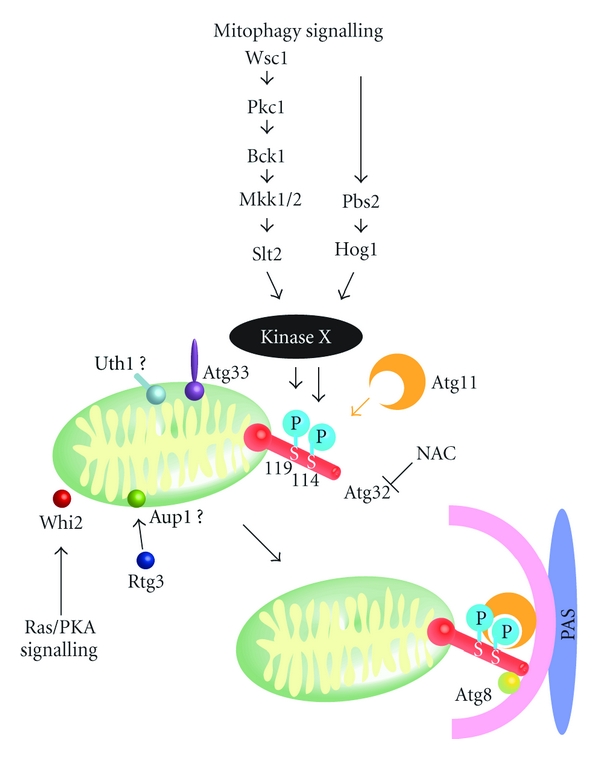
Mitophagy in yeast. Environmental or intracellular factors trigger the mitophagy-signaling pathways that include two MAPKs (Slt2 and Hog1), finally reaching and activating an unidentified kinase X. This kinase phosphorylates Ser114 and Ser119 on Atg32. Phosphorylation of Atg32, particularly at Ser114, mediates the Atg11–Atg32 interaction. Atg11 recruits mitochondria to the phagophore assembly site (PAS) where the autophagosome is generated to enclose the mitochondria. The antioxidant compound N-acetylcysteine (NAC) inhibits mitophagy, presumably by suppressing Atg32 expression. The Atg32–Atg8 interaction increases the efficiency of mitochondrial sequestration by the isolation membrane. Atg33, Whi2, Uth1, and Aup1 have been reported to be required for mitophagy. However, the function of these proteins in mitophagy has not been identified.

 We have summarized the above-described molecular processes and regulatory mechanisms in Figure 1.

## 4. The Physiological Role of Mitophagy in Yeast

 It has been suggested that mitophagy eliminates damaged or aged mitochondria, thereby maintaining mitochondrial quality. There are several lines of evidence demonstrating that damaged mitochondria are eliminated by mitophagy in yeast. Priault et al. suggested that conditional knockout of *fmc1*, a gene encoding the Fmc1 protein that is concerned with the folding of the F_1_F_o_-ATPase, induces mitophagy under anaerobic conditions [36]. Nowikovsky et al. suggested that interference with the mitochondrial K^+^/H^+^ exchanger Mdm38 causes the swelling of mitochondria and the degradation of those mitochondria by mitophagy [37]. Zhang et al. blocked mitochondrial DNA (mtDNA) replication using ethidium bromide or a mtDNA polymerase temperature-sensitive mutant and observed rapid degradation of mitochondria *via* autophagy [38]. These results indicate that mitochondrial damage is related to the induction of mitophagy, but are not direct evidence that autophagy selectively eliminates damaged mitochondria. Accordingly, it is still unknown whether mitophagy contributes to mitochondrial quality control in yeast. In fact, it has been difficult to identify the physiological role of mitophagy in yeast, because mitophagy-deficient *atg32*-deleted cells do not show any phenotype, including phenotypes of mitochondrial dysfunction [21].

 Our latest studies have partly revealed the physiological role of mitophagy in yeast. When mitophagy-deficient *atg32*-deleted cells were precultured in nonfermentable medium (for instance, lactate-containing medium as the sole carbon source) and were then shifted to nitrogen starvation for long-term culture (~5 days), the *atg32*-deleted cells grown on nutrient-rich plates generated small colonies, while wild-type cells did not. Further analysis revealed that, when wild-type cells encounter nitrogen starvation, they induce mitophagy and quickly eliminate mitochondria that have proliferated during respiratory growth. As a result, cellular ROS production, which occurs mainly in mitochondria, is suppressed. On the other hand, in mitophagy-deficient* atg32*-deleted cells, undegraded mitochondria produce excess ROS during nitrogen starvation. ROS damage mitochondria, and damaged mitochondria produce further ROS, finally leading to mtDNA deletion. Ultimately, cells with mtDNA deletion generate small colonies even in fermentable medium; this phenotype is called “petite” [39]. This suggests that mitophagy is required to regulate the number of mitochondria to minimize ROS production and, as a result, maintains the quality of mitochondria.

There have been several studies suggesting the interplay between mitochondria and autophagy. Bulk autophagy-deficient yeast strains exhibited reduced mitochondrial membrane potential, reduced activities of the electron transport chain, and higher levels of ROS and oxidative stress, resulting in the loss of mtDNA [38, 40]. In bulk autophagy-deficient cells, cellular ROS accumulate during nitrogen starvation because the cellular amino acid pool is reduced and the expression of the ROS scavenger proteins is suppressed [40]. This finding suggests that autophagy, including mitophagy, contributes to the quality control of mitochondria. In a contrasting situation, Graef and Nunnari demonstrated that healthy mitochondria are required for efficient induction of autophagy under amino acid starvation [41]. Autophagic flux is regulated by Atg1, target of rapamycin (TOR) kinase complex I, and cAMP-dependent protein kinase A (PKA), whereas *ATG8* induction is solely dependent on PKA. Defects in mitochondrial respiration induce PKA activity, resulting in the suppression of both *ATG8* induction and autophagic flux. Therefore, mitochondrial dysfunction directly affects and regulates autophagy. The data presented by Graef and Nunnari indicate that defects in mitochondrial respiration inhibit autophagy including mitophagy during amino acid starvation. They suggest that the effect of mitochondrial dysfunction on the regulation of autophagy varies according to the severity of the defect. Furthermore, these authors also suggest that inordinate accumulation of mitochondria that are defective in respiration beyond a certain level decreases the capacity for autophagy and mitophagy in these cells and evokes a negative feedback that results in cellular aging or death [41].

## 5. Studies of Mitophagy in Higher Eukaryotes

 As described above, the molecular processes and regulatory mechanisms of mitophagy in yeast have been slowly but surely identified. Since the 2008 report that a defect in mitophagy might be involved in the pathogenesis of Parkinson's disease [42], there has been much interest in mitophagy in higher eukaryotes and, in particular, mammalian cells. We will now summarize these studies.

### 5.1. Parkin/PINK1 and Mitophagy

**Figure 2:**
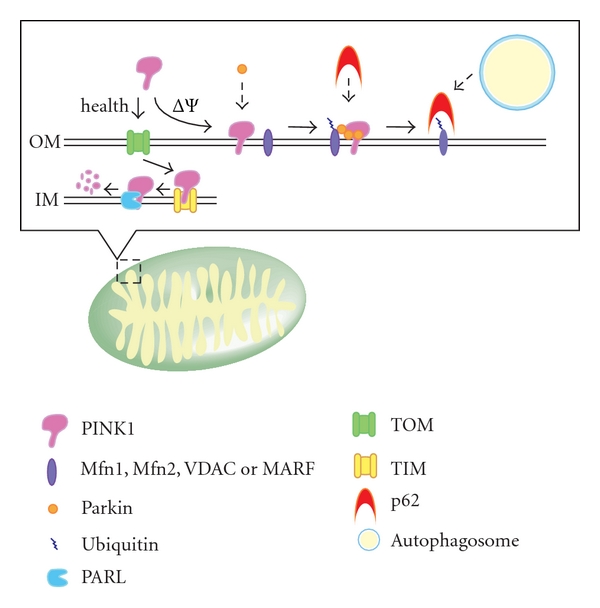
Parkin/PINK1 and mitophagy in higher eukaryotes. PINK1 is constitutively targeted and imported into the inner membrane *via *the mitochondrial import machinery, the TOM and TIM complexes, and degraded by presenilin-associated rhomboid-like protein (PARL). When the mitochondrial membrane potential is depolarized, PINK1 cannot translocate across the mitochondrial outer membrane and instead accumulates on it. PINK1 on the outer membrane causes the translocation of Parkin to mitochondria which triggers the ubiquitination of mitofusin 1 (Mfn1), mitofusin 2 (Mfn2), and voltage-dependent anion channel 1 (VDAC) in mammals, and mitochondrial assembly regulatory factor (MARF) in flies. The ubiquitinated proteins on mitochondria are captured by p62, a substrate of autophagy that can bind ubiquitinated proteins, resulting in the sequestration of mitochondria into autophagosomes. OM: outer membrane; IM: inner membrane.

Most mitophagy studies in mammalian cells have focused on PTEN-induced putative kinase protein 1 (PINK1)/Parkin-dependent mitochondrial degradation by autophagy. Parkin and PINK1 are encoded by the *PARK2* and *PARK6* genes, respectively; both are responsible for familial Parkinson's disease and have been reported to be associated with mitophagy [42–44]. PINK1 is expressed in the cytoplasm and constitutively translocates into the mitochondrial inner membrane where it is promptly degraded by the mitochondrial inner membrane rhomboid protease presenilin-associated rhomboid-like protein (PARL) [43, 45–47]. When mitochondria lose their membrane potential, PINK1 can target to the mitochondria, but cannot translocate across the mitochondrial outer membrane; therefore, it accumulates there. Accordingly, only depolarized mitochondria are marked by PINK1 accumulation. Parkin translocates to mitochondria in a PINK1-dependent manner [42–44, 48, 49]. Parkin triggers the ubiquitination of many mitochondrial proteins such as mitochondrial assembly regulatory factor (MARF) in flies or mitofusin 1, mitofusin 2, and voltage-dependent anion channel 1 (VDAC1) in mammalian cells [49–54]. The ubiquitinated proteins on mitochondria are bound by the autophagy substrate p62/SQSTM1, which contains a ubiquitin-associated domain, and the p62-associated mitochondria aggregate near the nucleus [49, 54, 55]. Because p62 is a substrate of autophagy, it is thought that p62-associated mitochondria are eventually degraded by autophagy [49, 54, 55]. We have summarized Parkin/PINK1-dependent mitophagy in Figure 2. Although it is accepted that p62 associates with mitochondrial proteins ubiquitinated by Parkin and mediates the aggregation of mitochondria, there have been conflicting reports showing that p62 is not indispensable for mitophagy [56, 57]. The histone deacetylase HDAC6, which can bind ubiquitinated proteins and facilitates the clearance of protein aggregates, is also reported to accumulate on mitochondria after Parkin translocation from the cytosol and mediate mitophagy [54]. Further studies are required to clarify the precise roles of p62 and HDAC6 in mitophagy.

### 5.2. Other Factors Related to Mitophagy in Higher Eukaryotes

 The study of Parkin/PINK1-dependent mitophagy has dominated the field in recent years. However, other studies have focused on Parkin/PINK1-independent mechanisms of mitophagy in higher eukaryotes.

 In mammalian cells, ULK1, a homolog of yeast Atg1, is known to be associated with the control of autophagy by the TOR signaling network. ULK1 activity is suppressed under nutrient-rich conditions by TOR complex 1 (TORC1) [58]. Recently, it has been suggested that phosphorylation of ULK1 by adenosine monophosphate-activated protein kinase (AMPK) is concerned with autophagy. Loss of AMPK or ULK1 resulted in deficient mitophagy and aggrephagy during starvation in mouse embryonic fibroblasts and hepatocytes, resulting in increases in the overall mitochondrial number and aberrant morphology [59]. This finding suggests that AMPK-mediated phosphorylation of ULK1 is required for mitochondrial homeostasis in nutrient-poor conditions.

 Tectonin domain-containing protein 1 (Tecpr1) has been identified as an Atg5-binding protein. This protein forms a complex with Atg12-Atg5-Atg16L1 and binds to WIPI-2, which is capable of association with phosphatidylinositol 3-phosphate at an isolation membrane. Interestingly, Tecpr1 is required for xenophagy, which selectively recognizes and eliminates bacterial pathogens such as *Shigella*, *Salmonella*, and Group A *Streptococcus*. Tecpr1 is also required for the autophagic degradation of misfolded protein aggregates and depolarized mitochondria but not for nonselective autophagy [60]. These findings suggest that Tecpr1 is an essential factor for specific cargo recognition in selective autophagy.

It has been reported that mitophagy is induced under several conditions such as mitochondrial permeability transition, during cellular development or during hypoxia. These three examples will be discussed in turn. First, nutrient starvation and photodamage, which both lead to mitophagy [12], cause mitochondrial permeability transition (MPT) [61], in which the opening of the MPT pores causes mitochondria to become permeable to all solutes up to a molecular mass of approximately 1500 Da, leading to mitochondrial depolarization and outer membrane rupture [62, 63]. Cyclosporin A, an inhibitor of MPT through interaction with cyclophilin D, blocks mitophagy during MPT [12, 64]. These findings suggest that MPT is a trigger for mitophagy that arises from mitochondria themselves.

 Second, recent studies have revealed that mitophagy plays an important role in cellular differentiation. During reticulocyte maturation (as with erythroid cell maturation mentioned in Section 1), mitochondria are eliminated *via* autophagy in a Nix-dependent manner [14, 17, 65]. Nix, in both *in vivo* and *in vitro* assays, interacts with LC3/GABARAP, which anchors to the isolation membrane and is involved in isolation membrane extension, and this Nix-LC3/GABARAP interaction is thought to mediate efficient targeting of mitochondria to autophagosomes [66, 67]. Similarly, when autophagy was inactivated by targeted deletion of the autophagy-essential gene *Atg7*, post-differentiated white adipocytes exhibited large numbers of mitochondria compared with the relatively few mitochondria observed in wild-type white adipocytes. This suggests that mitochondria are preferentially eliminated by autophagy during adipogenesis [18, 68–71].

 Third, mitophagy is induced by hypoxia in a Bcl-2/adenovirus E1B 19 kDa interacting protein 3-(BNIP3-) dependent manner; the expression of *BNIP3* is regulated by hypoxia-inducible factor [72–74]. This indicates that mitophagy might be a survival mechanism to regulate the production of ROS from mitochondria during hypoxia. As shown here, mitophagy plays a role in several aspects of cellular physiology, not just eliminating depolarized mitochondria in a Parkin/PINK1-dependent manner.

### 5.3. Unanswered Questions on Mitophagy in Mammalian Cells

 Although there are at present more than 50 publications regarding Parkin/PINK1-dependent mitophagy, the precise mechanisms are still unknown. Recently, it was reported that Parkin induces rupture of the outer membrane of depolarized mitochondria, depending on proteasomal activity, and then the ruptured mitochondria are eliminated by mitophagy [75]. This finding implies that Parkin and PINK1 are not the primary factors required for mitophagy but rather that they present depolarized mitochondria to the autophagic machineries by disrupting the mitochondrial outer membrane.

 Most of the autophagy-related genes identified in yeast are also present in mammals, suggesting that the molecular processes of autophagy are conserved throughout evolution. It is surprising then that the molecular processes of mitophagy and the essential factors identified to date are completely different between yeast and mammals. For example, in mammals, the mitochondrial receptor protein corresponding to Atg32 in yeast has not been identified.

## 6. Conclusion

 In recent years, there has been significant progress in studies of mitophagy in both yeast and mammals. In particular, the molecular processes and regulatory mechanisms of mitophagy in yeast have been well described, such as the specific Atg32–Atg11 interaction and the requirement for signaling by the two MAPKs Slt2 and Hog1. Meanwhile, the physiological role of mitophagy in mammalian cells has been well understood. Because mitophagy is evolutionarily conserved, it is reasonable to speculate that there will be similar molecular processes, regulatory mechanisms, and physiological roles in both yeast and mammals. The interplay of yeast and mammalian mitophagy studies will consolidate our understanding of this cellular process.

## References

[1] Berg TO, Fengsrud M, Strømhaug PE, Berg T, Seglen PO (1998). Isolation and characterization of rat liver amphisomes: evidence for fusion of autophagosomes with both early and late endosomes. *Journal of Biological Chemistry*.

[2] Dunn WA (1990). Studies on the mechanisms of autophagy: formation of the autophagic vacuole. *Journal of Cell Biology*.

[3] Nakatogawa H, Suzuki K, Kamada Y, Ohsumi Y (2009). Dynamics and diversity in autophagy mechanisms: lessons from yeast. *Nature Reviews Molecular Cell Biology*.

[4] Yang Z, Klionsky DJ (2010). Eaten alive: a history of macroautophagy. *Nature Cell Biology*.

[5] Mizushima N, Yamamoto A, Hatano M (2001). Dissection of autophagosome formation using Apg5-deficient mouse embryonic stem cells. *Journal of Cell Biology*.

[6] Rubinsztein DC (2006). The roles of intracellular protein-degradation pathways in neurodegeneration. *Nature*.

[7] Mizushima N, Levine B, Cuervo AM, Klionsky DJ (2008). Autophagy fights disease through cellular self-digestion. *Nature*.

[8] Levine B, Mizushima N, Virgin HW (2011). Autophagy in immunity and inflammation. *Nature*.

[9] Clark SL (1957). Cellular differentiation in the kidneys of newborn mice studies with the electron microscope. *The Journal of Biophysical and Biochemical Cytology*.

[10] Takeshige K, Baba M, Tsuboi S, Noda T, Ohsumi Y (1992). Autophagy in yeast demonstrated with proteinase-deficient mutants and conditions for its induction. *Journal of Cell Biology*.

[11] Twig G, Elorza A, Molina AJA (2008). Fission and selective fusion govern mitochondrial segregation and elimination by autophagy. *EMBO Journal*.

[12] Kim I, Rodriguez-Enriquez S, Lemasters JJ (2007). Selective degradation of mitochondria by mitophagy. *Archives of Biochemistry and Biophysics*.

[13] Kim I, Lemasters JJ (2011). Mitophagy selectively degrades individual damaged mitochondria after photoirradiation. *Antioxidants and Redox Signaling*.

[14] Schweers RL, Zhang J, Randall MS (2007). NIX is required for programmed mitochondrial clearance during reticulocyte maturation. *Proceedings of the National Academy of Sciences of the United States of America*.

[15] Sandoval H, Thiagarajan P, Dasgupta SK (2008). Essential role for Nix in autophagic maturation of erythroid cells. *Nature*.

[16] Nishida Y, Arakawa S, Fujitani K (2009). Discovery of Atg5/Atg7-independent alternative macroautophagy. *Nature*.

[17] Zhang J, Randall MS, Loyd MR (2009). Mitochondrial clearance is regulated by Atg7-dependent and -independent mechanisms during reticulocyte maturation. *Blood*.

[18] Zhang Y, Goldman S, Baerga R, Zhao Y, Komatsu M, Jin S (2009). Adipose-specific deletion of *autophagy-related gene 7 (atg7)* in mice reveals a role in adipogenesis. *Proceedings of the National Academy of Sciences of the United States of America*.

[19] Kiššova I, Salin B, Schaeffer J, Bhatia S, Manon S, Camougrand N (2007). Selective and non-selective autophagic degradation of mitochondria in yeast. *Autophagy*.

[20] Okamoto K, Kondo-Okamoto N, Ohsumi Y (2009). Mitochondria-anchored receptor Atg32 mediates degradation of mitochondria via selective autophagy. *Developmental Cell*.

[21] Kanki T, Wang K, Cao Y, Baba M, Klionsky DJ (2009). Atg32 is a mitochondrial protein that confers selectivity during mitophagy. *Developmental Cell*.

[22] Shintani T, Huang WP, Stromhaug PE, Klionsky DJ (2002). Mechanism of cargo selection in the cytoplasm to vacuole targeting pathway. *Developmental Cell*.

[23] Farré JC, Manjithaya R, Mathewson RD, Subramani S (2008). PpAtg30 tags peroxisomes for turnover by selective autophagy. *Developmental cell*.

[24] Kanki T, Klionsky DJ (2008). Mitophagy in yeast occurs through a selective mechanism. *Journal of Biological Chemistry*.

[25] Kanki T, Wang K, Baba M (2009). A genomic screen for yeast mutants defective in selective mitochondria autophagy. *Molecular Biology of the Cell*.

[26] Mao K, Wang K, Zhao M, Xu T, Klionsky DJ (2011). Two MAPK-signaling pathways are required for mitophagy in *Saccharomyces cerevisiae*. *Journal of Cell Biology*.

[27] Aoki Y, Kanki T, Hirota Y (2011). Phosphorylation of serine 114 on Atg32 mediates mitophagy. *Molecular Biology of the Cell*.

[28] Manjithaya R, Jain S, Farré JC, Subramani S (2010). A yeast MAPK cascade regulates pexophagy but not other autophagy pathways. *Journal of Cell Biology*.

[29] Kanki T, Wang K, Klionsky DJ (2010). A genomic screen for yeast mutants defective in mitophagy. *Autophagy*.

[30] Tal R, Winter G, Ecker N, Klionsky DJ, Abeliovich H (2007). Aup1p, a yeast mitochondrial protein phosphatase homolog, is required for efficient stationary phase mitophagy and cell survival. *Journal of Biological Chemistry*.

[31] Mendl N, Occhipinti A, Müller M, Wild P, Dikic I, Reichert AS (2011). Mitophagy in yeast is independent of mitochondrial fission and requires the stress response gene *WHI2*. *Journal of Cell Science*.

[32] Kissova I, Deffieu M, Manon S, Camougrand N (2004). Uth1p is involved in the autophagic degradation of mitochondria. *Journal of Biological Chemistry*.

[33] Müller M, Reichert AS (2011). Mitophagy, mitochondrial dynamics and the general stress response in yeast. *Biochemical Society Transactions*.

[34] Journo D, Mor A, Abeliovich H (2009). Aup1-mediated regulation of Rtg3 during mitophagy. *Journal of Biological Chemistry*.

[35] Deffieu M, Bhatia-Kiššová I, Salin B, Galinier A, Manon S, Camougrand N (2009). Glutathione participates in the regulation of mitophagy in yeast. *Journal of Biological Chemistry*.

[36] Priault M, Salin B, Schaeffer J, Vallette FM, di Rago JP, Martinou JC (2005). Impairing the bioenergetic status and the biogenesis of mitochondria triggers mitophagy in yeast. *Cell Death and Differentiation*.

[37] Nowikovsky K, Reipert S, Devenish RJ, Schweyen RJ (2007). Mdm38 protein depletion causes loss of mitochondrial K^+^/ H^+^ exchange activity, osmotic swelling and mitophagy. *Cell Death and Differentiation*.

[38] Zhang Y, Qi H, Taylor R, Xu W, Liu LF, Jin S (2007). The role of autophagy in mitochondria maintenance: characterization of mitochondrial functions in autophagy-deficient *S. cerevisiae* strains. *Autophagy*.

[39] Kurihara Y, Kanki T, Aoki Y (2012). Mitophagy plays an essential role in reducing mitochondrial production of reactive oxygen species and mutation of mitochondrial DNA by maintaining mitochondrial quantity and quality in yeast. *The Journal of Biological Chemistry*.

[40] Suzuki SW, Onodera J, Ohsumi Y (2011). Starvation induced cell death in autophagy-defective yeast mutants is caused by mitochondria dysfunction. *PLoS ONE*.

[41] Graef M, Nunnari J (2011). Mitochondria regulate autophagy by conserved signalling pathways. *EMBO Journal*.

[42] Narendra D, Tanaka A, Suen DF, Youle RJ (2008). Parkin is recruited selectively to impaired mitochondria and promotes their autophagy. *Journal of Cell Biology*.

[43] Narendra DP, Jin SM, Tanaka A (2010). PINK1 is selectively stabilized on impaired mitochondria to activate Parkin. *PLoS Biology*.

[44] Vives-Bauza C, Zhou C, Huang Y (2010). PINK1-dependent recruitment of Parkin to mitochondria in mitophagy. *Proceedings of the National Academy of Sciences of the United States of America*.

[45] Jin SM, Lazarou M, Wang C, Kane LA, Narendra DP, Youle RJ (2010). Mitochondrial membrane potential regulates PINK1 import and proteolytic destabilization by PARL. *Journal of Cell Biology*.

[46] Deas E, Plun-Favreau H, Gandhi S (2011). PINK1 cleavage at position A103 by the mitochondrial protease PARL. *Human Molecular Genetics*.

[47] Shi G, Lee JR, Grimes DA (2011). Functional alteration of PARL contributes to mitochondrial dysregulation in Parkinson's disease. *Human Molecular Genetics*.

[48] Matsuda N, Sato S, Shiba K (2010). PINK1 stabilized by mitochondrial depolarization recruits Parkin to damaged mitochondria and activates latent Parkin for mitophagy. *Journal of Cell Biology*.

[49] Geisler S, Holmström KM, Skujat D (2010). PINK1/Parkin-mediated mitophagy is dependent on VDAC1 and p62/SQSTM1. *Nature Cell Biology*.

[50] Ziviani E, Tao RN, Whitworth AJ (2010). *Drosophila* Parkin requires PINK1 for mitochondrial translocation and ubiquitinates Mitofusin. *Proceedings of the National Academy of Sciences of the United States of America*.

[51] Poole AC, Thomas RE, Yu S, Vincow ES, Pallanck L (2010). The mitochondrial fusion-promoting factor mitofusin is a substrate of the PINK1/parkin pathway. *PLoS ONE*.

[52] Gegg ME, Cooper JM, Chau KY, Rojo M, Schapira AHV, Taanman JW (2010). Mitofusin 1 and mitofusin 2 are ubiquitinated in a PINK1/parkin-dependent manner upon induction of mitophagy. *Human Molecular Genetics*.

[53] Tanaka A, Cleland MM, Xu S (2010). Proteasome and p97 mediate mitophagy and degradation of mitofusins induced by Parkin. *Journal of Cell Biology*.

[54] Lee JY, Nagano Y, Taylor JP, Lim KL, Yao TP (2010). Disease-causing mutations in Parkin impair mitochondrial ubiquitination, aggregation, and HDAC6-dependent mitophagy. *Journal of Cell Biology*.

[55] Ding WX, Ni HM, Li M (2010). Nix is critical to two distinct phases of mitophagy, reactive oxygen species-mediated autophagy induction and Parkin-ubiquitin-p62-mediated mitochondrial priming. *Journal of Biological Chemistry*.

[56] Okatsu K, Saisho K, Shimanuki M (2010). P62/SQSTM1 cooperates with Parkin for perinuclear clustering of depolarized mitochondria. *Genes to Cells*.

[57] Narendra DP, Kane LA, Hauser DN, Fearnley IM, Youle RJ (2010). p62/SQSTM1 is required for Parkin-induced mitochondrial clustering but not mitophagy; VDAC1 is dispensable for both. *Autophagy*.

[58] Chan EY (2009). MTORC1 phosphorylates the ULK1-mAtg13-FIP200 autophagy regulatory complex. *Science Signaling*.

[59] Egan DF, Shackelford DB, Mihaylova MM (2011). Phosphorylation of ULK1 (hATG1) by AMP-activated protein kinase connects energy sensing to mitophagy. *Science*.

[60] Ogawa M, Yoshikawa Y, Kobayashi T (2011). A Tecpr1-dependent selective autophagy pathway targets bacterial pathogens. *Cell Host and Microbe*.

[61] Lemasters JJ, Qian T, Elmore SP (1998). Confocal microscopy of the mitochondrial permeability transition in necrotic cell killing, apoptosis and autophagy. *BioFactors*.

[62] Zoratti M, Szabo I (1995). The mitochondrial permeability transition. *Biochimica et Biophysica Acta*.

[63] Forte M, Bernardi P (2005). Genetic dissection of the permeability transition pore. *Journal of Bioenergetics and Biomembranes*.

[64] Rodriguez-Enriquez S, Kai Y, Maldonado E, Currin RT, Lemasters JJ (2009). Roles of mitophagy and the mitochondrial permeability transition in remodeling of cultured rat hepatocytes. *Autophagy*.

[65] Kundu M, Lindsten T, Yang CY (2008). Ulk1 plays a critical role in the autophagic clearance of mitochondria and ribosomes during reticulocyte maturation. *Blood*.

[66] Schwarten M, Mohrlüder J, Ma P (2009). Nix directly binds to GABARAP: a possible crosstalk between apoptosis and autophagy. *Autophagy*.

[67] Novak I, Kirkin V, McEwan DG (2010). Nix is a selective autophagy receptor for mitochondrial clearance. *EMBO Reports*.

[68] Baerga R, Zhang Y, Chen PH, Goldman S, Jin S (2009). Targeted deletion of *autophagy-related 5 (atg5)* impairs adipogenesis in a cellular model and in mice. *Autophagy*.

[69] Goldman S, Zhang Y, Jin S (2010). Autophagy and adipogenesis: implications in obesity and type II diabetes. *Autophagy*.

[70] Goldman Scott SJ, Taylor R, Zhang Y, Jin S (2010). Autophagy and the degradation of mitochondria. *Mitochondrion*.

[71] Goldman SJ, Zhang Y, Jin S (2011). Autophagic degradation of mitochondria in white adipose tissue differentiation. *Antioxidants and Redox Signaling*.

[72] Zhang H, Bosch-Marce M, Shimoda LA (2008). Mitochondrial autophagy is an HIF-1-dependent adaptive metabolic response to hypoxia. *Journal of Biological Chemistry*.

[73] Band M, Joel A, Hernandez A, Avivi A (2009). Hypoxia-induced BNIP3 expression and mitophagy: *in vivo* comparison of the rat and the hypoxia-tolerant mole rat, *Spalax ehrenbergi*. *FASEB Journal*.

[74] Zhang J, Ney PA (2009). Role of BNIP3 and NIX in cell death, autophagy, and mitophagy. *Cell Death and Differentiation*.

[75] Yoshii SR, Kishi C, Ishihara N, Mizushima N (2011). Parkin mediates proteasome-dependent protein degradation and rupture of the outer mitochondrial membrane. *Journal of Biological Chemistry*.

